# In Vitro Propagation of Rheophytic Orchid, *Epipactis flava* Seidenf.—A Comparison of Semi-Solid, Continuous Immersion and Temporary Immersion Systems

**DOI:** 10.3390/biology8040072

**Published:** 2019-09-24

**Authors:** Boworn Kunakhonnuruk, Phithak Inthima, Anupan Kongbangkerd

**Affiliations:** Plant Tissue Culture Research Unit, Department of Biology, Faculty of Science, Naresuan University, Phitsanulok 65000, Thailand

**Keywords:** liquid medium, *Epipactis flava*, rheophytic orchid, conservation

## Abstract

*Epipactis flava* Seidenf. is an endangered Thai rheophytic orchid that has recently shown a rapid decrease in its natural habitat, prompting an urgent need for conservation using ex situ reintroduction methods. Temporary immersion system (TIS) has been successfully applied for large-scale propagation in various plants species. Propagation efficiency of *E. flava* using TIS was investigated and compared with conventional semi-solid system (SSS) and liquid continuous immersion system (CIS). The highest percentage of new shoot and shoot bud formation was obtained from TIS, followed by CIS and SSS, respectively. Growth parameters as indicated by number of new shoots, shoot buds, shoot height and leaves per explant were significantly higher using TIS than with SSS and CIS. Moreover, the maximum number of new shoots and shoot buds per replication were reliably obtained from TIS higher than SSS and CIS. After acclimatization, the highest survival percentage of plantlets was observed in TIS (76.7%), with 60% surviving after eight weeks of transplantation in artificial stream. TIS was determined as the most suitable culture system for in vitro mass propagation of *E. flava* compared to CIS and SSS.

## 1. Introduction

A rheophytic lifestyle appears to be very rare in Orchidaceae, and the only rheophytic orchid found in Thailand is *Epipactis flava* [1]. Intricate morphological details and life cycle of *E*. *flava* were described by Pedersen et al. [2] and Kunakhonnuruk et al. [3], respectively. In Thailand, *E. flava* grows in streams with calcareous substrates [2]. Presently, only 10 natural sites of *E*. *flava* have been found in Nan, Kanchanaburi and Tak Provinces [1] and its current conservation status is classified as an endangered species [4,5]. Loss of natural habitat due to erosion and human activity have accelerated the rate of population decline. Urgent action is now required for *E*. *flava* conservation. Plant tissue culture is recognized as a high-performance tool for ex situ conservation of endemic and endangered orchid species [6,7,8]. Recently, successful in vitro seed germination and seedling culture of *E. flava* was reported [3]. However, for in vitro mass propagation, conventional techniques using semi-solid or shake-flask cultures are labor intensive during the subculturing period. To overcome these problems, bioreactor systems have been developed and improved. Recently, several novel bioreactor systems have shown promise, including temporary immersion system (TIS), which are recognized as a key process for mass propagation and commercial exploitation of plant tissue cultures [9]. Moreover, TIS and other novel plant bioreactors based on micropropagation have been used to increase plant culture multiplication rates and successfully applied commercially [10,11,12] for mass production of medicinal plants [13,14] and for conservation of endangered species [8]. During in vitro culture conditions, plants are grown under the high relative humidity, low light intensity, constant temperature—in contrast to ex vitro environment. Hence, intensive acclimatization and transplantation of plantlets or seedlings from the in vitro to ex vitro environment was necessary. This new research technology offers the possibility of large-scale *E. flava* cultivation. Greenhouse acclimatization of in vitro derived plantlets was investigated as well to establish a complete propagation program applicable for species reintroduction or ex situ conservation.

## 2. Materials and Methods

### 2.1. Evaluation of Different In Vitro Culture Systems

The germination of *E. flava* seeds obtained from green pod was performed under aseptic condition in 120 mL culture vessel containing 20 mL of semi-solid medium. After few months, in vitro seedlings of *E. flava* with two to three leaves were cultured on modified Murashige and Skoog medium [15] supplemented with 150 mL L^−1^ coconut water (CW), 50 g L^−1^ potato extract (PE) and 20 g L^−1^ sucrose [3] for two months before being used as explants. Explants of *E. flava* with two to three buds differentiated from rhizomes that were cultured in semi-solid system (SSS), continuous immersion system (CIS) and TIS using the same medium. The SSS system was performed with a 120 mL of bottle holding 20 mL of medium solidified with 7.5 g L^−1^ agar and 2 g L^−1^ activated charcoal, whereas CIS was conducted with a 125 mL conical flask containing 20 mL liquid medium and shaken on a 110 rpm rotary shaker. One explant per vessel was performed for SSS and CIS. Each culture system consists of three replications and each replication contain 20 vessels. A TIS system modified from Escalona et al. [16] was set up as well using 1 L of twin-bottle supported with glass beads for the cultivating bottle. Twenty explants were cultured in a TIS vessel containing 400 mL liquid medium. One TIS set represented one replicate, and three replicates were set up for this system. The liquid medium in TIS was fed for 5 min every 4 h. All culture systems were maintained under 12/12 h light/dark photoperiods with 40 µmol m^−2^ s^−1^ light intensity using a warm-white LED lamp (BIOLED-SET-18W, BioBULB^®^, United Digital Corporation CO., LTD., Bangkok, Thailand) for four weeks. Survival rate, plant quality and quantity were observed to evaluate propagation efficiency of the different culture systems.

### 2.2. Greenhouse Acclimatization and Ex Vitro Culture

Plantlets of *E. flava* from the different culture systems were used for acclimatization and ex vitro cultivation. In vitro plantlets were rinsed with running tap water to eliminate the culture medium. Subsequently, they were individually transplanted into plastic slit pots (4 cm diameter, 4 cm depth) containing mixed potting medium; Hydroton (4 mm diameter; POPPER, Wiwan Technology, Chiang Mai, Thailand): Pumice (5 mm diameter; Lombok Pumice Stone, Semarang, Indonesia) (1:1). After that, plantlets containing in plastic slit pots were placed in a transparent plastic box (36 × 50 × 14 cm) for nine weeks of acclimatization. The plantlets were watered once a week and sprayed with liquid N-P-K fertilizer (1 g L^−1^) (20-20-20; WESCO chemicals Thailand CO., LTD., Bangkok, Thailand) every two weeks. After nine weeks of acclimatization, the plantlets were transferred for cultivation in an artificial stream for a further eight weeks under a greenhouse environment (ambient temperature 27 to 32 °C, relative humidity 50% to 60% and natural sunlight with an average of 250 µmol m^−2^ s^−1^ light intensity under 70% shade nets). Survival rate and plant quality were evaluated and recorded.

### 2.3. Statistical Analysis

Complete randomized design was employed. Differences of each parameter among the various culture systems were statistically compared by one-way ANOVA followed by Duncan’s new Multiple Range Test (DMRT) using SPSS program ver. 17.0 (SPSS^®^, New York, NY, USA).

## 3. Results

### 3.1. Growth and Development of E. flava Plantlets Under Different In Vitro Culture Systems

**Figure 1:**
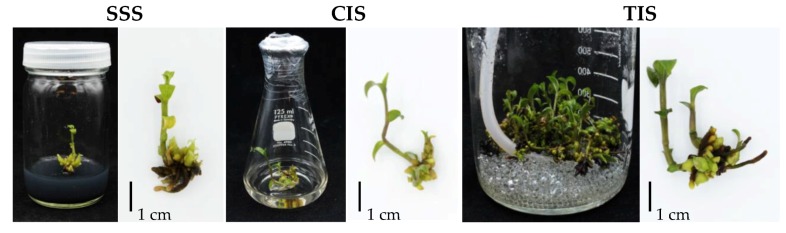
Growth and development of *Epipactis flava* plantlets at four weeks after culture in semi-solid system (**SSS**), continuous immersion system (**CIS**) and temporary immersion system (**TIS**).

**Figure 2:**
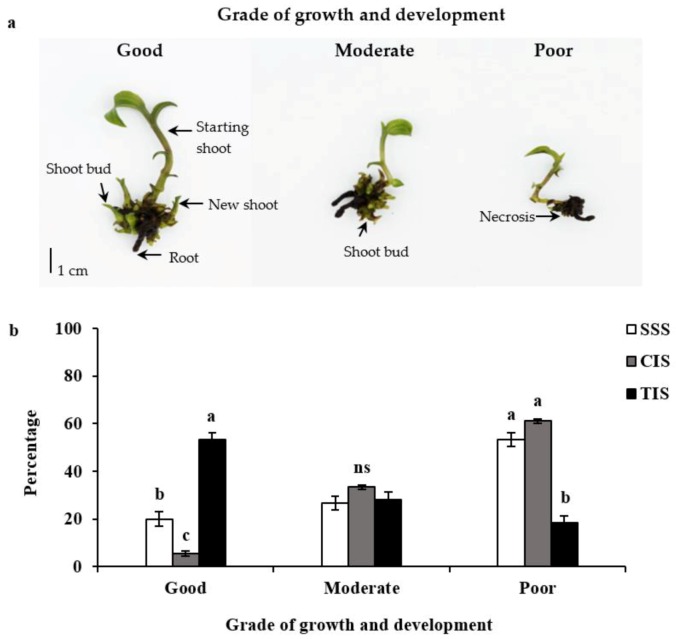
Classifying system for growth of *Epipactis flava* plantlets (**a**) and comparative effects of culture systems on growth of *Epipactis flava* plantlets after four weeks of culture (**b**). Results represent the mean ± SE of three replications from 20 explants. Different letters within the same grade of growth were significantly different at *p* ≤ 0.05 according to Duncan’s Multiple Range Test (DMRT).

Propagation efficiencies of *E. flava* in SSS, CIS and TIS were evaluated and compared. One hundred percent survival rates of plantlets were found in all culture systems. Explants from the tested culture systems grew well and some proliferated new shoots and shoot buds (Figure 1). No hyper hydric symptoms were observed in the explants after four weeks of culture. Regeneration and proliferation efficiency of explants grown in the three culture systems showed obvious differences. Quality of regenerated plantlets was classified into three levels, as illustrated in Figure 2a. Results revealed the highest percentage of healthy growing plantlets in TIS (53.3%) followed by SSS (20.0%) and CIS (5.5%), respectively (Figure 2b). More than 50% of poor-quality plantlets were observed in both SSS and CIS. TIS showed higher positive influence on growth and development of *E. flava* than SSS and CIS (Table 1). The highest percentages of new shoot formation (96.7%) and shoot bud formation (91.7%) were observed in TIS followed by SSS (46.7%) and CIS (40.0%), respectively. In addition, TIS promoted the growth of new shoots (1.5 shoots per explant), shoot buds (8.1 shoot buds per explant) and roots (4.4 roots per explant) and stimulated shoot height (29.4 mm per shoot) and number of leaves (4.4 leaves per shoot) more than SSS and CIS as well. Orchid shoot heights in TIS were up to two-folds higher than in SSS and CIS. A comparison of total number of new shoots and shoot buds obtained from one replication (20 explants per replication) revealed that the TIS system resulted in significantly higher amounts of both new shoots (29.3) and shoot buds (161.0) than SSS and CIS systems (Table 1).

### 3.2. Acclimatization and Ex Vitro Culture of Plantlets Derived From Different In Vitro Culture Systems

**Figure 3:**
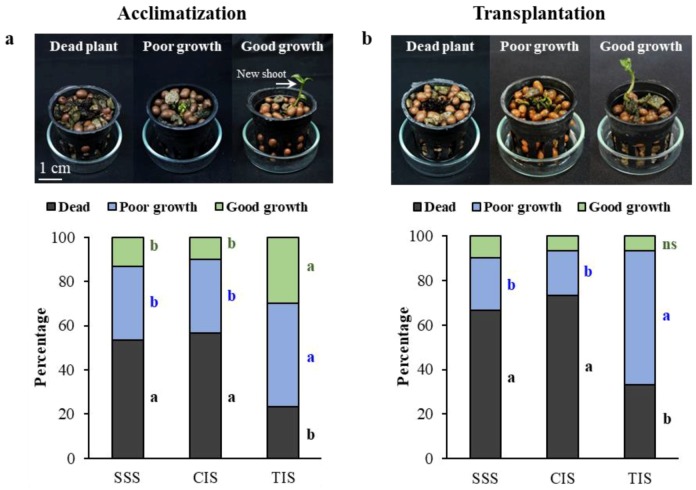
Plant quality and growth of *Epipactis flava* after acclimatization for nine weeks (**a**) and after transplantation within an artificial stream for eight weeks (**b**). Results are the mean of three replications (20 plants per replication). The same letter of each observation parameter was not significantly different at *p* ≤ 0.05 according to DMRT.

Survival and growth of *E. flava* plantlets obtained from different in vitro culture systems were investigated under acclimatization condition. Complete hardening was established at nine weeks. Different plant qualities of *E. flava* were evaluated according to the following criteria: dead plant; poor growth plant, survived plant with no new shoots or shoot bud formation; good growth plant and survived plant with new shoots or shoot bud formation (Figure 3a). The highest survival rate was observed in plantlets obtained from TIS (76.7%) whereas less than 50% of plantlets derived from SSS and CIS survived after acclimatization (Figure 3a). In addition, most plants showing good growth (38.9%) were found in TIS-derived plantlets followed by plantlets from SSS (28.9%) and CIS (23.3%), respectively. The last cultivation step of *E. flava* plantlets was performed after acclimatization with transfer to an artificial stream under a greenhouse environment for eight weeks. Results revealed that *E. flava* plantlets obtained from TIS had approximately two-fold higher survival rates (60%) during cultivation in an artificial stream than plantlets from SSS and CIS (Figure 3b).

## 4. Discussion

Advances in in vitro culture techniques such as TIS have recently been developed to improve mass propagation efficiency that was difficult to accomplish using conventional propagation systems such as SSS or CIS [17]. TIS mass propagation success has previously been reported for various plant species [18,19]—including orchids [11,20,21]. Prior reports revealed that different culture systems resulted in diverse growth and morphogenesis of cultured plants [11,14,22]. The highest ratio of normal plantlets was obtained in TIS treatment, whereas SSS and CIS methods exhibited higher plant-abnormality rate [18,23]. The SSS medium possibly provided less nutrient absorption than TIS and CIS [16], while CIS frequently induced abnormality and necrosis symptoms resulting from permanent immersion [24]. Better growth and proliferation of *E. flava* plants cultured by CIS over SSS were reported by Kunakhonnuruk et al. [3]; however, no differences were observed under shorter culturing time. Therefore, large-scale mass production of *E. flava* by CIS was not recommended, although this plant generally displays a rheophytic habit. Results indicated that TIS was a more suitable method for large-scale mass production of this endangered orchid, with better growth and proliferation of *E. flava* than SSS and CIS. Undesirable symptoms as explained by browning appearance at the root were found in *E. flava* cultured under TIS lower than SSS and CIS. Furthermore, TIS showed better shoot and shoot bud proliferation as well as root and leaf induction numbers compared to SSS and CIS. Relatively similar results were found in *Vanilla planifolia* [25] and *Rubus* spp. as well [26]. One advantage of TIS over SSS and CIS is the alteration between aeration and periodical immersion of explants in a liquid medium which improves gaseous exchange, increases oxygen supply and reduces hyperhydration [27,28,29]. Furthermore, TIS eliminates some toxic gases as well, i.e., ethylene, during air feeding immersion cycles [30], whereas air ventilation is not an option in closed systems such as SSS and CIS. Photomixotrophic culture was enhanced during renewal of atmosphere in TIS which stimulated better growth and development of *E. flava* than SSS and CIS.

Acclimatization is an important procedure to support successful plantlet transplantation from the in vitro to ex vitro environment [31,32]. This process allows in vitro plantlets to survive and adapt to the natural environment which normally has higher light intensity and lower humidity than in vitro conditions [31]. During the acclimatization process, plantlets of *E. flava* obtained from TIS gave higher survival rates and successfully grew and adapted under environmental change. By contrast, morphological plantlet disorders were mostly derived from SSS and CIS. Many previous reports indicated that plantlets from TIS had greater growth, with rapid adaptation after exposure to ex vitro conditions than SSS and CIS derived plant [18,33]. This might be because TIS enhanced stomatal functioning and improved photosynthesis and transpiration [31,34]. However, *E. flava* plantlets visibly wilted during transfer to ex vitro acclimatization. Related reports found that wilting symptoms caused by temporary dormancy during transplantation could occur in some species, i.e., *Eulophia cullenii* [35] and *Calopogon tuberosus* [36]. Plantlets of *E. flava* from all culture systems were transferred at the acclimatization step to cultivation in artificial streams for a further eight weeks. Results revealed that TIS derived plantlets had higher survival percentage than those from SSS and CIS. Therefore, to promote the reintroduction program of this endangered orchid, TIS offers a suitable in vitro culture system for the continuous mass production of healthy plantlets within a short time and rapidly encourages acclimatized plantlets to natural environments.

## 5. Conclusions

This is the first study comparing TIS for mass propagation of *E. flava* against CIS and SSS. TIS was found to be the most efficient and suitable method for mass propagation of *E. flava*. Plantlets obtained from TIS successfully adapted and had the highest survival rate during acclimatization and *ex vitro* culture. TIS significantly improved mass propagation and offers advantages for ex situ conservation of *E. flava*.

## Figures and Tables

**Table 1:** Effect of culture systems on growth and development of *Epipactis flava* plantlets for four weeks of culture.

Parameter	Culture Systems ^1^		
	SSS	CIS	TIS
Survival rate (%)	100.0 ± 0.0 ns	100.0 ± 0.0	100.0 ± 0.0
New shoot formation (%)	86.7 ± 0.6 b	70.0 ± 1.1 c	96.7 ± 1.3 a
Number of new shoots per explant	1.0 ± 0.0 b	0.8 ± 0.2 b	1.5 ± 0.1 a
Number of new shoots per replication	19.0 ± 1.0 b	15.7 ± 1.5 b	29.3 ± 8.6 a
Shoot bud formation (%)	46.7 ± 1.3 b	40.0 ± 1.1 b	91.7 ± 1.7 a
Number of shoot buds per explant	3.9 ± 0.1 b	5.5 ± 0.2 b	8.1 ± 0.4 a
Number of shoot buds per replication	78.7 ± 5.5 b	110.7 ± 14.6 b	161.0 ± 35.6 a
Number of roots per explant	3.8 ± 0.0 ab	3.6 ± 0.0 b	4.4 ± 0.1 a
Number of leaves per shoot ^2^	2.8 ± 0.1 b	2.7 ± 0.5 b	4.4 ± 0.1 a
Shoot height (mm) ^2^	14.0 ± 0.4 b	14.3 ± 3.4 b	29.4 ± 0.8 a

Values are mean ± SE of three replications (20 explants per replication) except number of new shoot and shoot bud per replication are mean ± SD of three replications. The same letter within a row was not significantly different at *p* ≤ 0.05 according to DMRT. ^1^ SSS—Semi-solid system; CIS—Continuous immersion system and TIS—Temporary immersion system. ^2^ Number of leaves per shoot and shoot height were recorded from the longest shoot of each explant.
